# Motor-sparing peripatellar plexus block provides noninferior block duration and complete block area of the peripatellar region compared with femoral nerve block: a randomized, controlled, noninferiority study

**DOI:** 10.1186/s12871-022-01863-7

**Published:** 2022-11-01

**Authors:** Wen-Yi Gong, Chen-Guang Li, Jing-Yu Zhang, Xiao-Hui Liao, Cheng Zhu, Jie Min, Xiao-Fang Yue, Kun Fan

**Affiliations:** 1 Department of Anaesthesiology, Wusong Central Hospital, Shanghai, China; 2 Department of Anaesthesiology, First People’s Hospital of Tianshui , Gansu, China; 3 Department of Anaesthesiology, Second Hospital Affiliated to Lanzhou University, Gansu, China; 4 Department of Orthopaedics, Wusong Central Hospital, Shanghai, China; 5 Department of Neurology, Shanghai Sixth People’s Hospital, No. 600, Yishan Road, 200233 Shanghai, China; 6 Department of Anaesthesiology, Shanghai Sixth People’s Hospital, No. 600, Yishan Road, 200233 Shanghai, China

**Keywords:** Peripatellar plexus, Femoral nerve, Knee surgery, Nerve block, Ultrasound

## Abstract

**Background:**

Developing adequate regional anaesthesia for knee surgeries without affecting lower limb mobilization is crucial to perioperative analgesia. However, reports in this regard are limited. We proposed a technique for ultrasound-guided peripatellar plexus (PP) block. Compared with the femoral nerve (FN) block, we hypothesized that this technique would provide a noninferior block duration and a complete cutaneous sensory block in the peripatellar region without affecting lower limb mobilization. An investigation was conducted to verify our hypothesis in cadavers and volunteers.

**Methods:**

The study was designed in two parts. First, eight cadaveric lower limbs were dissected to verify the feasibility of PP block after methylene blue injection under ultrasound. Second, using a noninferiority study design, 50 healthy volunteers were randomized to receive either a PP block (PP group) or an FN block (FN group). The primary outcome was the duration of peripatellar cutaneous sensory block, with the prespecified noninferiority margin of -3.08 h; the secondary outcome was the area of peripatellar cutaneous sensory block; in addition, the number of complete anaesthesias of the incision line for total knee arthroplasty and the Bromage score 30 min after block were recorded.

**Results:**

The PP was successfully dyed, whereas the FN and saphenous nerve were unstained in all cadaveric limbs. The mean difference of the block duration between the two groups was − 1.24 (95% CI, -2.81 − 0.33) h, and the lower boundary of the two-sided 95% CI was higher than the prespecified noninferiority margin (P_noninferiority_ = 0.023), confirming the noninferiority of our technique over FN block. The cutaneous sensory loss covered the entire peripatellar region in the PP group. PP block achieved complete anaesthesia of the incision line used for total knee arthroplasty and a Bromage score of 0 in 25 volunteers, which differed significantly from that of volunteers who underwent FN block.

**Conclusion:**

Ultrasound-guided PP block is a feasible technique. Compared with FN block, PP block provides noninferior block duration and complete blocking of the peripatellar region without affecting lower limb mobilization.

**Trial registration:**

This study was registered in the Chinese Clinical Trial Register (registration no. ChiCTR2000041547, registration date 28/12/2020).

## Background

Perioperative analgesia for knee surgeries including total knee arthroplasty (TKA), knee arthroscopy, patellar surgery, and superficial knee surgery, remains difficult to manage [1–3]. Peripheral nerve block is a common method used to achieve pain control after knee surgeries [1], among which femoral nerve (FN) block is classically used for postoperative analgesia [2–4]. However, FN block alone only provides sensation to the anteromedial aspect of the knee [5], and quadriceps inhibition caused by FN block will increase the incidence of postoperative complications and prolong the hospital stay.

The most common approach for knee surgeries is the anterior approach in the peripatellar region. Nociceptors are widely distributed throughout the superficial tissue of the peripatellar region and are associated with moderate to severe pain in knee surgeries using the anterior approach [6, 7]. Complete block of the superficial tissue in the peripatellar region is key for controlling moderate to severe pain without affecting lower limb mobilization in knee surgeries using the anterior approach. However, reports in this regard are limited.

The skin and subcutis covering the peripatellar region are innervated by the peripatellar plexus (PP), a subcutaneous network of communicating nerve fibres that travel over and around the patella consisting of the inferior patellar branch of the saphenous nerve (IPBSN), the anterior branch of the medial femoral cutaneous nerve (aMFCN), the intermediate femoral cutaneous nerve (IFCN) originating from the FN, and anterior branch of the lateral femoral cutaneous nerve (aLFCN) [8]. We speculated that the block duration of the PP block would be similar to that of the FN block but that the block area of the PP block would be different from that of the FN block in peripatellar region. In addition, it is unclear whether the spread of local anaesthetics from an injection at the PP would involve the motor nerves such as the FN.

**Fig. 1:**
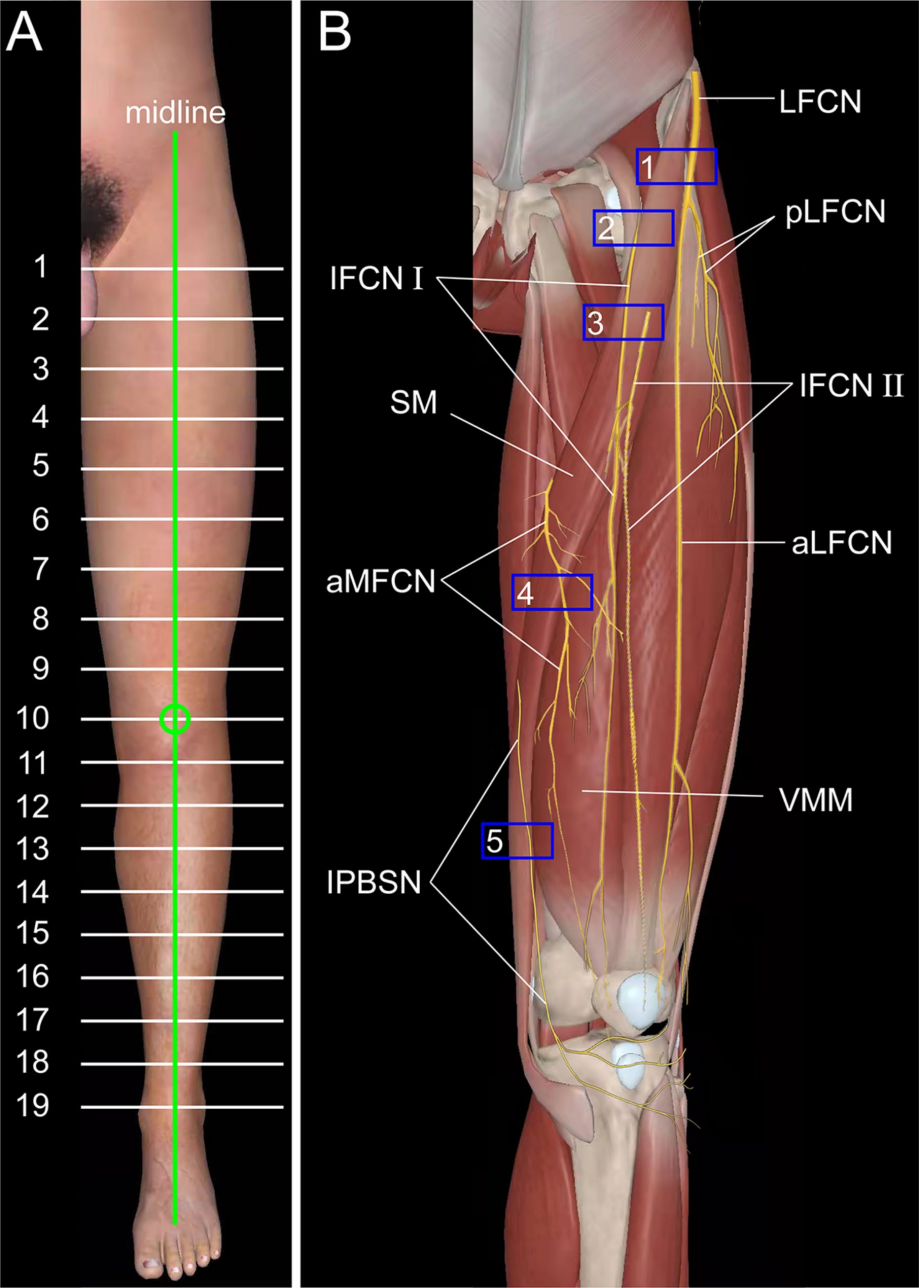
**A** Schematic diagram of 19 planes along the midline for the lower limbs, 10 equal planes for the thigh and 10 for the crus; **B** Schematic diagram of the probe position for ultrasound-guided PP block. SM, sartorius muscle; VMM, vastus medialis muscle; LFCN, lateral femoral cutaneous nerve; aLFCN, anterior branch of lateral femoral cutaneous nerve; pLFCN, posterior branch of the lateral femoral cutaneous nerve; IPBSN, infrapatellar branch of the saphenous nerve; IFCN I;, intermediate femoral cutaneous nerve branch I;; IFCN II;, intermediate femoral cutaneous nerve branch II;; aMFCN, anterior branch of the medial femoral cutaneous nerve. Green circles represent the centre of the patella; blue box 1 represents the probe position for the LFCN block; blue box 2 represents the probe position for the IFCN I; block; blue box 3 represents the probe position for the IFCN II; block; blue box 4 represents the probe position for the aMFCN block; and blue box 5 represents the probe position for the IPBSN block

Our previous publications have described the block technique for IPBSN, aMFCN and IFCN (Fig. 1B) [9–11]. The aLFCN is a branch of the lateral femoral cutaneous nerve (LFCN) which can be identified and blocked under ultrasound (Fig. 1B) [12]. Hence, PP block under ultrasound is theoretically feasible. We previously performed PP block in a few cadavers and volunteers, and our results indicated that this technique might be feasible [13]. However, further study is needed to confirm these findings.

Given these issues, the purpose of the present study was to verify the feasibility of the ultrasound-guided PP block by injecting dye solution and identifying the anatomic course of these nerves in cadavers, and to compare the analgesic characteristics of PP block versus FN block with a randomized, controlled noninferiority trial. The primary outcome was the block duration, and the secondary outcome was the block area in the peripatellar region.

## Methods

### Cadaveric study

This study was approved by the Ethics Committee of Wusong Hospital (approval no. 2020‒Y‒18). Eight lower limbs of four lightly embalmed cadaveric specimens ranging in age from 69 to 80 yrs were obtained through the Department of Anatomy, Fudan University. None of the cadavers exhibited any evidence of gross pathology, previous surgical procedures, or traumatic lesions. Ultrasound-guided injection of 0.01% methylene blue (diluted in normal saline) was performed using a high-frequency linear transducer (6‒13 MHz, SonoSite SII, Washington, USA).

### LFCN injection

**Fig. 2:**
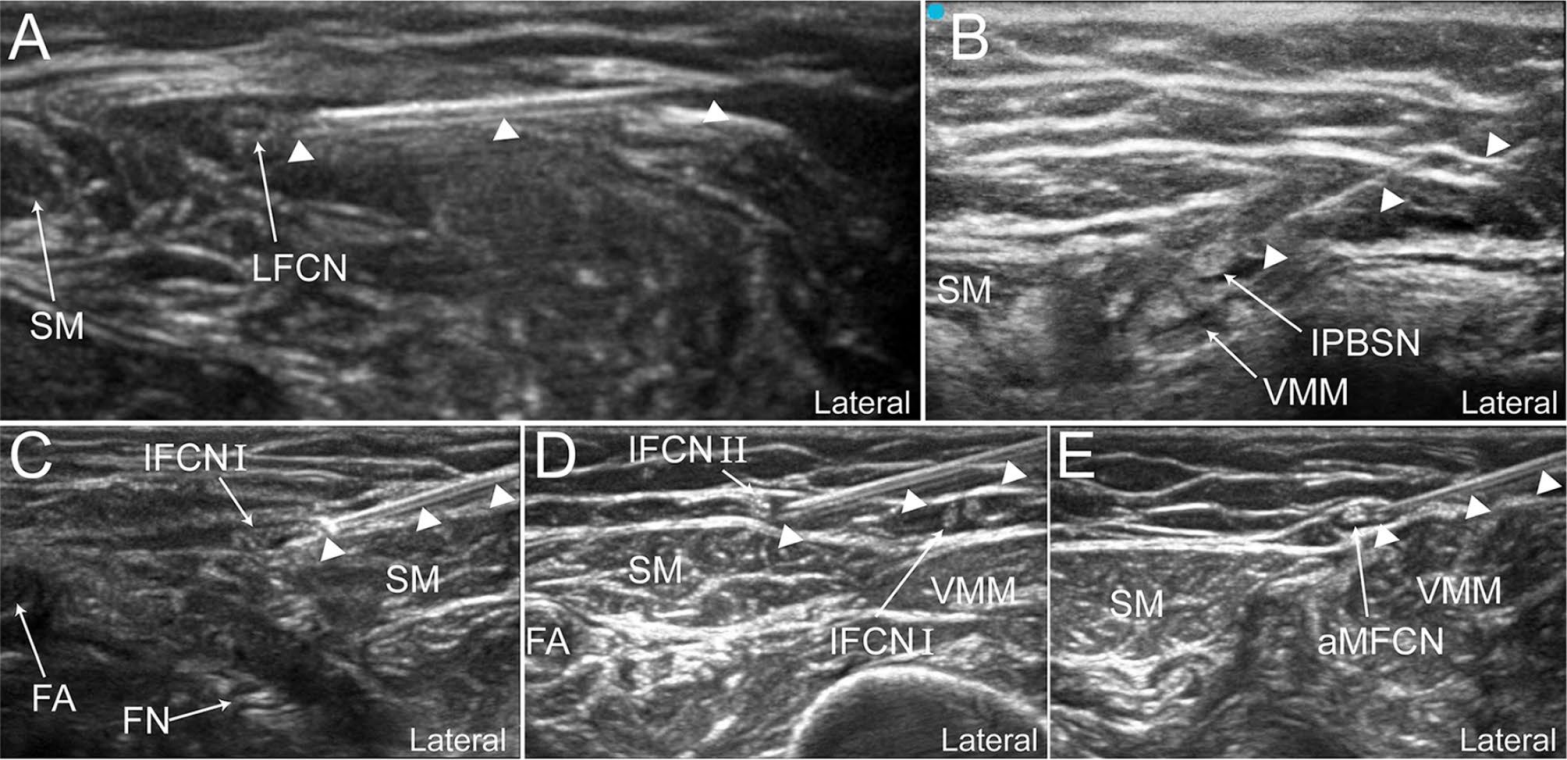
Ultrasound-guided dye injection of the PP in cadavers. **A** Dye injection of the LFCN; **B** Dye injection of the IPBSN; **C** Dye injection of the IFCN I; **D**, Dye injection of the IFCN II; **E** Dye injection of the aMFCN. SM, sartorius muscle; VMM, vastus medialis muscle; FA, femoral artery; FN, femoral nerve; LFCN, lateral femoral cutaneous nerve; IPBSN, infrapatellar branch of the saphenous nerve; IFCN I, intermediate femoral cutaneous nerve branch I; IFCN II, intermediate femoral cutaneous nerve branch II; aMFCN, anterior branch of the medial femoral cutaneous nerve. White triangles represent the needle trajectory

The aLFCN injection was replaced by the LFCN injection between the tensor fasciae latae and the sartorius muscle (SM) with 5 ml of dye solution as previously described (Figs. 1B and 2 A) [12].

### IPBSN injection

The IPBSN injection was performed close to the proximal medial femoral epicondyle where the IPBSN is superficial to the junction of the SM and the vastus medialis muscle (VMM), using 5 ml of dye solution (Figs. 1B and 2B) [10].

### IFCN injection

The IFCN I and IFCN II injections were carried out at the medial margin of the SM and superficial to the SM, respectively (Figs. 1B and 2 C, 2D) [11]. A perineural injection of 2.5 ml of dye solution was performed for both nerves.

### aMFCN injection

The aMFCN injection was performed on the medial median segment of the thigh where the aMFCN is superficial to the junction of the SM and VMM (Figs. 1B and 2E) [9]. A perineural injection of 5 ml of dye solution was performed.

The abovementioned procedures were performed by two anaesthesiologists who played no further role in the study.

### Data collection

Thirty minutes after the injections, the specimen was dissected by two anatomists who played no further role in the study. The anatomic course of the PP and the staining of the four cutaneous nerves were evaluated. In addition, the staining of the saphenous nerve (SN) in the adductor canal (AC) and the FN was assessed.

### Volunteer Study

#### Study design and registration

This prospective, single-centre, randomized, parallel controlled noninferiority trial was approved by the Ethics Committee of Wusong Hospital, China (approval no. 2020‒Y‒17). This study was registered in the Chinese Clinical Trial Register (registration no. ChiCTR2000041547, registration date 28/12/2020). The present report followed the Consolidated Standards of Reporting Trials (CONSORT) guidelines.

#### Recruitment

Volunteers were recruited between 1 and 15 January 2021. Written informed consent was provided prior to study commencement. The trial was performed at the block room of Wusong Hospital from 20 January to 10 February 2021. Volunteers of either sex with age ≥ 18 year were eligible for recruitment. The exclusion criteria were psychiatric disorders; inability to cooperate or communicate in Chinese; chronic pain; ingestion of any pain medication; lower limb neuropathy; and contraindications for regional anaesthesia (coagulopathy, local infection at the block site, allergy to local anaesthetic).

#### Randomization and blinding

The volunteers were randomly assigned to either the PP group or the FN group using a computer-generated randomization sequence at a 1:1 ratio by a statistician who was otherwise not involved in the study. The allocation results were concealed in sealed opaque and sequentially numbered envelopes that were provided to the research coordinator. On the day of nerve block, the research coordinator provided one envelope per subject to the anaesthesiologist who performed the block procedures. To eliminate performance bias as far as possible, all blocks were performed by four experienced regional anaesthesiologists who played no further role in the study: two performing PP block and the other two performing FN block. The volunteers, research coordinator, orthopaedic experts and statistician were blinded to group allocation.

#### Preparation for nerve block

All volunteers had intravenous access established in the forearm and underwent standard monitoring of heart rate, noninvasive blood pressure, and oxygen saturation. The nondominant lower limb was selected for study. The midline of the thigh was drawn from the centre of the patella perpendicular to the horizontal line at the medial end of the inguinal crease. Similarly, the midline of the crus was drawn from the centre of the patella to the level of the ankle. The thigh and the crus were each divided into 10 equal planes along the midline for 19 planes in total (Fig. 1 A).

**Fig. 3:**
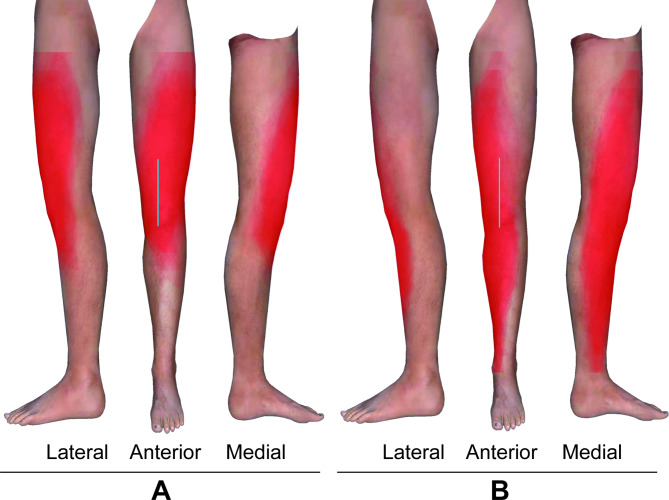
Superimposed images of the block areas in the lower limbs (standard left lower limbs) of the two groups. **A** PP group; **B** FN group. The block areas are shown in red. The blue line is the incision line for TKA.

Two orthopaedic experts marked the incision line for TKA on the lower limbs to be blocked in each volunteer. The incision line for TKA was drawn from 5 cm proximal to the base of patella to approximately 1 cm medial to the tibial tuberosity (Fig. 3) [14].

#### Ultrasound-guided nerve block

In the PP group, PP block was performed as described in the cadaveric study using 5 ml of 0.33% ropivacaine for the LFCN, aMFCN, and IPBSN and 2.5 ml of 0.33% ropivacaine for both the IFCN I and IFCN II. In the FN group, in accordance with previous studies, FN block was performed by injecting 20 ml of 0.33% ropivacaine [15].

#### Outcome measurements

All assessments and data collections were performed by the research coordinator. The primary outcome was the block duration. The pinprick cutaneous sensation test was performed to assess the block duration using a 24-G needle twice with an interval of approximately 1 s between individual stimulations. The absence of pain and tactile sensation to both stimuli was assessed as cutaneous sensory block. The test was performed every half hour from the end of the injection of the local anaesthetics. The block duration was defined as the time interval from the beginning of the cutaneous sensory block to the beginning of the cutaneous sensation recovery at the anaesthetized skin in the patellar region.

The secondary outcome was the block area in the peripatellar region. The borders of cutaneous sensory loss were marked with dots on the skin at each of the 19 abovementioned planes 30 min after block. The distances from the medial and lateral dots to the vertical centreline were measured and calculated as percentages of the semicircumference. According to the result of this calculation, the dots showing the borders of medial and lateral cutaneous sensory loss were marked on the 19 planes of the “standard leg” using Adobe Photoshop CC 2019 software (Adobe Systems Software Ireland Limited, Dublin, Ireland). Then, the dots were connected to generate a block area in the “standard leg” and filled with red. The brightness of each volunteer’s scaled cutaneous sensory loss map was 4%. The block areas were overlapped one by one on two identical “standard legs” in accordance with the different groups. The overlapping areas became darker in colour with each added volunteer, enabling identification of the cutaneous sensory areas of the leg that were most likely to be blocked (Fig. 3).

In addition, the number of volunteers with complete anaesthesia of the incision line for TKA, the onset time of block (defined as the time interval from the end of the injection of the local anaesthetic to the earliest appearance of a decrease in the pinprick sensation at the anaesthetized skin in the patellar region), the Bromage score (grade 0 = free movement of leg and feet; 1 = only able to flex knees with free movement of the feet; 2 = unable to flex knees, free movement of feet; 3 = unable to move the legs or feet [16]) of the affected lower limb 30 min after the nerve block, and nerve block-related complications (vascular injury, haematoma, and local anaesthetic intoxication), were recorded.

#### Sample size calculation

The sample size was calculated based on the primary outcome according to the noninferiority hypothesis using PASS 15 software. Based on our pilot study, the mean block duration in the PP group and the FN group was 14.06 and 15.38 h, respectively; the SD of the mean block duration in the PP group and the FN group was 2.41 h and 1.81 h, respectively. The noninferiority margin was set as -3.08 h (20% of the mean duration of cutaneous sensory block in the FN group [17]). With two-sided α of 0.05 and power of 80%, 24 volunteers were required in each group. Considering a potential dropout rate of 5% in the follow-up data collection, we planned to include 25 volunteers in each group.

### Statistical analysis

The noninferiority hypothesis for the primary outcome was tested using the two-sided t test, and the mean difference (MD) with corresponding two-sided 95% confidence interval (CI) was calculated. Noninferiority was determined when the lower boundary of the two-sided 95% CI was higher than − 3.08 h (P_non−inferiority_ < 0.05 was considered to indicate statistical significance).

The normality of the distribution of continuous data was examined with Kolmogorov-Smirnov test. The variance homogeneity was examined by Levene test. Data with a normal distribution and homogeneity of variance were presented as the mean ± SD, and the two groups were compared using Student’s t test. The MD with the corresponding two-sided 95% CI was calculated. Categorical data were presented as numbers (%). Unordered categorical data were compared using Pearson’s χ² test. The relative risk (RR) with corresponding two-sided 95% CI was calculated. Ordered categorical data were compared using Mann-Whitney test. Two-tailed tests were performed whenever appropriate, and P values of < 0.05 were considered to indicate statistically significance.

The IBM SPSS Statistics for software for Windows (version 22.0, SPSS Inc., USA) was used for all statistical analyses.

## Results

### Cadaveric study

**Fig. 4:**
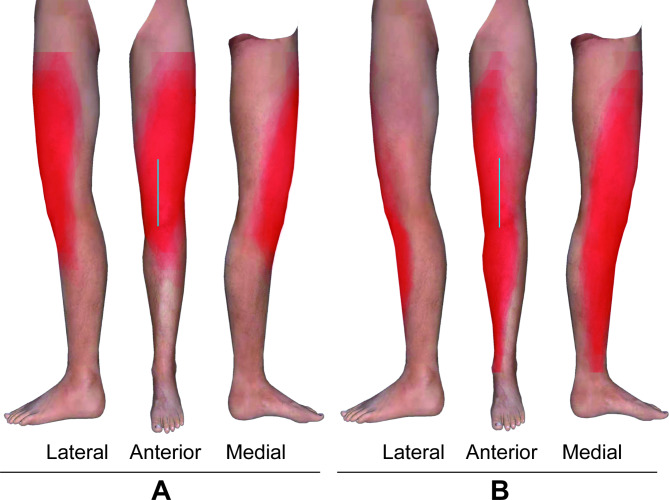
Course and staining of the peripatellar plexus 30 min after dye injection. **A** Course and staining of the LFCN; **B** Course and staining of the IPBSN; **C** Course and staining of the IFCN; **D** Course and staining of the aMFCN; **E** Staining of the FN. ASIS, anterior superior iliac spine; SM, sartorius muscle; aLFCN, anterior branch of the lateral femoral cutaneous nerve; pLFCN, posterior branch of the lateral femoral cutaneous nerve; IPBSN, infrapatellar branch of the saphenous nerve; IFCN I, intermediate femoral cutaneous nerve branch I; IFCN II, intermediate femoral cutaneous nerve branch II; aMFCN, anterior branch of the medial femoral cutaneous nerve; SN, saphenous nerve; FN, femoral nerve

The PP was clearly identified by ultrasound in all eight lower limbs of the four cadavers (Fig. 2). The dye injection and dissection were successfully completed in all lower limbs. The aLFCN was stained with methylene blue in all eight lower limbs, whereas the posterior branches of the LFCN were stained in six of the eight lower limbs (Fig. 4 A, C). In all lower limbs, the IPBSN ramified from the SN in the AC followed by piercing of the SM, and was stained on the surface of the SM, while the SN was unstained (Fig. 4B, D). In all lower limbs, IFCN I ran along the medial margin of the SM then superficial to the SM, while IFCN II pierced and then ran superficial to the SM. Both IFCN I and IFCN II were stained on the surface of the SM in all lower limbs (Fig. 4 C, E). The aMFCN followed the medial margin of the SM in seven of eight lower limbs; in the remaining lower limb, the aMFCN pierced and ran medial to the SM. The aMFCN was completely stained medial to the SM in the median inferior segment of the thigh in all lower limbs (Fig. 4 A, D). The FN was unstained in any cadaveric limbs (Fig. 4E).

### Volunteer study

**Fig. 5:**
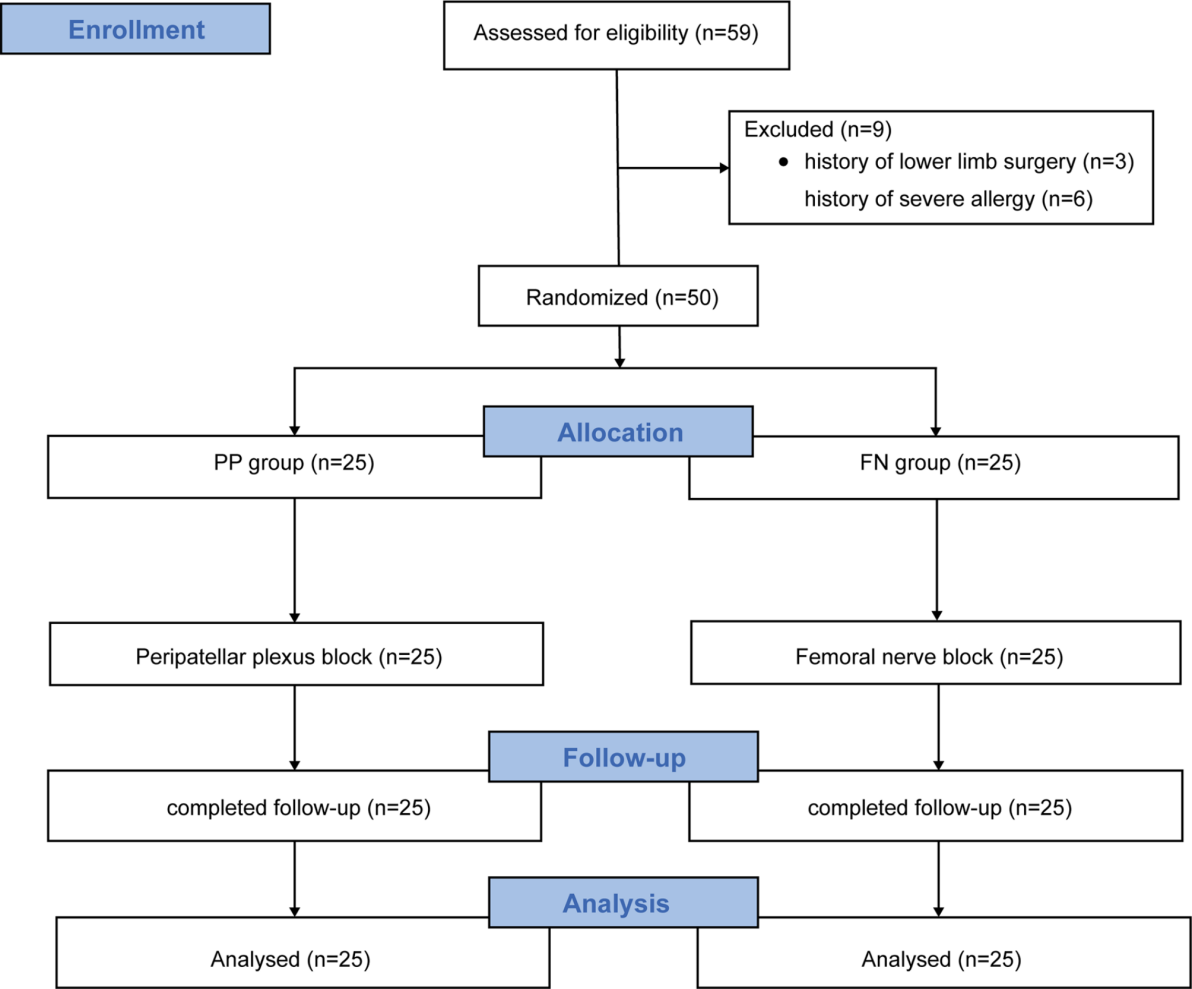
Consolidated Standards of Reporting Clinical Trials flow diagram. The sample size is indicated in parentheses. PP, peripatellar plexus; FN, femoral nerve

Whilst n = 59 volunteers were screened as potentially suitable, n = 50 met the inclusion and exclusion criteria and were randomized into the two groups (Fig. 5). PP block or FN block was successfully performed in all volunteers and no volunteers were lost to follow-up (Fig. 5). The baseline demographic characteristics of the volunteers in the two study groups were similar, with no statistically significant differences (Table 1). No volunteers had nerve block-related complications.

**Table 1:** Baseline characteristics of volunteers

Parameter	PP group (n = 25)	FN group (n = 25)	P value
Age (years)	48.96 ± 16.69	45.64 ± 13.21	0.439^a^
Sex, male/female	11/14 (44%/56%)	9/16 (36%/64%)	0.564^b^
Body mass index (kg/m2)	25.76 ± 3.11	24.19 ± 3.43	0.097^a^
Height (cm)	165.24 ± 9.64	163.44 ± 7.74	0.470^a^
Crus length (cm)	36.88 ± 2.73	36.56 ± 2.47	0.666^a^
Thigh length (cm)	30.76 ± 3.24	29.90 ± 2.58	0.305^a^
Side, left/right	13/11 (52%/48%)	9/16 (36%/64%)	0.254^b^

Data are expressed as mean ± SD for continuous outcomes; and as number (%) for categorical outcomes. PP, peripatellar plexus; FN, femoral nerve; a, Student’s t test; b, Pearson’s χ² test

**Fig. 6:**
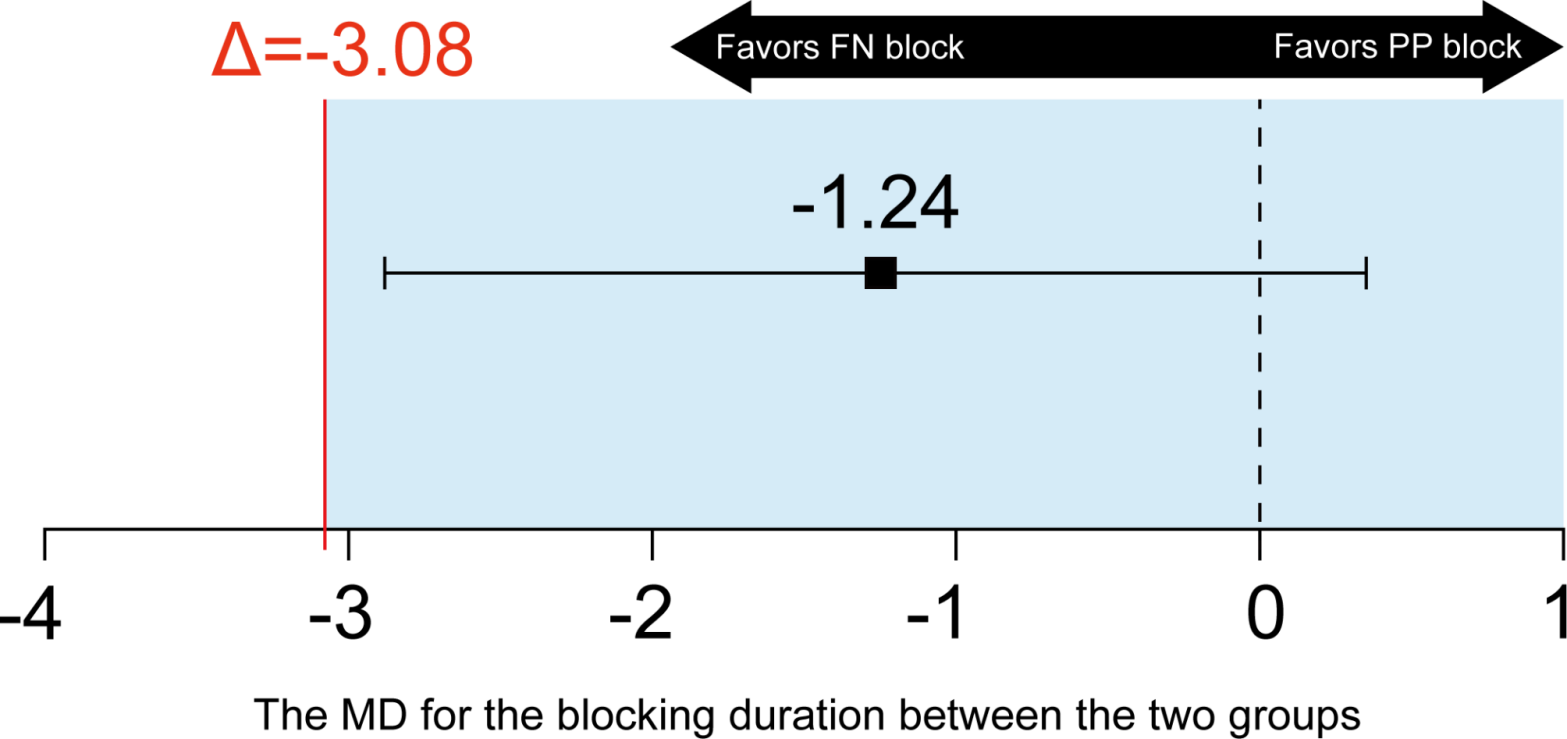
Noninferiority diagram of MD for the block duration between the PP group and FN group. PP, peripatellar plexus; FN, femoral nerve. Error bars indicate two-sided 95% CI of the MD between the two groups. The solid red line indicates a noninferiority margin (Δ) of -3.08. The shaded region indicates the zone of noninferiority

In the assessment of the primary outcome, the block duration was 14.72 ± 2.50 h in the PP group and 15.96 ± 3.01 h in the FN group. The MD for the block duration between the two groups was − 1.24 (95% CI, -2.81 − 0.33; P = 0.119) h. Because the lower boundary of the two-sided 95% CI was higher than the prespecified noninferiority margin (Δ = -3.08 h), noninferiority was established (P_noninferiority_ = 0.023; Fig. 6; Table 2).

**Table 2:** Nerve block characteristics in the two groups

Outcomes	PP group (n = 25)	FN group (n = 25)	Estimate (95% CI)	P value
The block duration (h)	14.72 ± 2.50	15.96 ± 3.01	MD: -1.24 (-2.81, 0.33)	0.119^a^
The onset time of block (min)	2.00 ± 2.00	1.62 ± 1.02	MD: 0.38 (-0.52, 1.28)	0.402^a^
Complete anesthesia of the incision line for TKA	25 (100%)	14 (56%)	RR: 1.79 (1.26, 2.53)	0.001^b^
Bromage scores 30 min after block			NA	< 0.001^c^
0	25 (100%)	0 (0%)		
1	0 (0%)	12 (48%)		
2	0 (0%)	13 (52%)		
3	0 (0%)	0 (0%)		

Data are expressed as mean ± SD and MD (95% CI) for continuous outcomes; and as number (%) and RR (95% CI) for categorical outcomes. MD, mean difference; RR: relative risk; CI, confidence interval; NA, not applicable; PP, peripatellar plexus; FN, femoral nerve; a, Student’s t test; b, Pearson’s χ² test.; c, Mann-Whitney test

Regarding the secondary outcome measured, the brightness of the overlapping colour was dark and consistent in the entire peripatellar region in the PP group, whereas the brightness of the overlapping colour was light and inconsistent in the anterior, superior and lateral peripatellar regions in the FN group, indicating that the cutaneous sensory block in the PP group covered the entire peripatellar region, whereas that in the FN group did not. (Fig. 3)

In addition, the onset time of the block did not differ between the two groups (MD, 0.38; 95% CI, -0.52 − 1.28; P = 0.402; Table 2). Complete anaesthesia of the incision line used for TKA was achieved in all 25 volunteers in the PP group versus 14 of 25 volunteers in the FN group (RR, 1.79; 95% CI, 1.26 − 2.53; P = 0.001; Table 2). The Bromage score 30 min after block was 0 in all 25 volunteers in the PP group; in the FN group, the Bromage score 30 min after block was more than 0 in all 25 volunteers (P < 0.001; Table 2).

## Discussion

Nociceptors are abundant throughout the superficial tissue of the peripatellar region and are thus strongly associated with moderate to severe pain in knee surgeries [6, 7]. Adjunct use of an IPBSN block is associated with significant pain relief after anterior cruciate ligament repair for 16‒24 h [18]. The addition of anterior femoral cutaneous nerve (AFCN, including the IFCN and MFCN) block to femoral triangle block (FTB) decreases the opioid requirement compared to that of FTB alone after TKA [19]. These studies indicated that anaesthesia for the superficial tissue of the knee facilitates postoperative analgesia after knee surgery. Our results demonstrated that PP block could cover the entire peripatellar region. Hence, PP block might be of great importance in pain management after knee surgery.

A previous study showed that FN block alone could maintain anaesthesia for 4‒6 h and postoperative analgesia for 6‒30 h; these ranges are due to differences in the surgeries, the anaesthetics used and the concentrations of local anaesthetics [20]. Our results from the volunteer trial suggested that the onset of PP block was rapid, and the duration of sensory block after PP blockade was 13 h, which was noninferior to that after FN block. This suggests that PP block might achieve a similar duration of postoperative analgesia to FN block for some superficial knee surgeries.

Although FN block is a standard analgesic intervention following knee surgery, such as TKA and anterior cruciate repair [21], the analgesia is limited to the anteromedial aspect of the knee [5]. Our results demonstrated that the anterior, superior and lateral peripatellar regions were not covered after FN block in some volunteers, and the incision line for TKA in 44% of volunteers in the FN group was not completely blocked. This finding might account for the suboptimal analgesia from FN block for certain knee surgeries. Conversely, PP block covered the entire peripatellar region, and the incision line of TKA was completely blocked in all volunteers of the PP group. All of the results above suggest that PP block is a better option for analgesia of the superficial tissue in the peripatellar region than FN block and might be more suitable for perioperative analgesia in some knee surgeries.

Although several techniques for PP block have been reported previously, none of them can block the four nerves of the PP completely or cover the entire peripatellar region [22, 23]. It was recently reported that a combination of FTB with IFCN block could cover most of the peripatellar region [24]. However, regarding pain after TKA, the lateral patellar region cannot be anaesthetized due to the inability to block the LFCN [24]. A volunteer study reported that AFCN block could not achieve complete anaesthesia of the incision line for TKA even combined with FTB and would even reduce maximal knee extension force [22]. Another published study showed that blocking several of the PP branches could not achieve complete anaesthesia of the entire peripatellar region [23]. Previous studies have also demonstrated that the IFCN, aMFCN, IPBSN, and aLFCN cross-innervate the peripatellar region anatomically, resulting in a poor block effect and incomplete block area when only one is blocked [9–12]. These results suggest that all four nerves constituting the PP should be blocked if a complete cutaneous sensory block of the peripatellar region is to be achieved.

Current studies of PP focusing on either anatomy or ultrasound have examined only some of the four nerves of PP and have fallen short in terms of details, impeding the development and clinical application of PP block. A cadaver study under ultrasound confirmed the feasibility of block for several PP branches, but this study demonstrated a poor success rate with IPBSN block and did not provide details on identifying among the MFCN, IFCN I and IFCN II under ultrasound [25]. Another cadaveric study under ultrasound identified only the MFCN and one branch of the IFCN, with no detailed identification of the PP branches [26]. We found that the IFCN, aMFCN and IPBSN could be easily distinguished using the SM as a marker. This was confirmed to be an effective approach by both the present study and our previous study.

Poor postoperative mobilization of the lower limb not only limits the patient’s ability to participate early in any physical rehabilitation program, but also places the patient at risk for falling and thrombosis [27]. The results of our volunteer study showed that FN block caused a reduction in muscle strength of the lower limb after the block. A study of anterior cruciate ligament reconstruction showed that the quadriceps strength returns approximately 13.1 h after FN block, adversely affecting postoperative rehabilitation after surgery [28]. The results of our volunteer study showed that PP block did not affect lower limb mobilization. The dissection in this study also showed that neither the FN nor the AC was involved when injecting to the PP, which demonstrated that PP block did not affect lower limb mobilization. Therefore, PP block may be more conducive to early functional exercise after knee surgery.

AC block is commonly used in perioperative analgesia for knee surgeries because it minimizes motor blockade compared with FN block. However, the quadriceps weakness associated with AC block is not negligible [27, 29] because it can prevent functional rehabilitation postoperatively. Moreover, AC block may be inadequate for analgesia for TKA [30]. AC block cannot achieve complete anaesthesia of the skin and subcutis in the peripatellar region, and it is ineffective for articular pain as well [24]. Compared with AC block, the blocked area produced following PP block may be larger in the peripatellar region. When combined with block of the sensory nerves supplying the knee joint capsule, including interspace between the popliteal artery and the capsule of the posterior knee (IPACK) block or genicular nerve block, PP block may also be superior to AC block for knee surgery. Further comparative studies are required to confirm this theory.

The PP innervates the sensory areas of the skin and subcutis covering the peripatellar region, as well as the sensory areas of the partial knee capsule and internal structures of the knee joint [31, 32]. Hence, PP block might be applied not only for anaesthesia of superficial surgeries in the peripatellar region but also for analgesia of deep knee surgeries. However, for deep knee surgeries, including TKA and cruciate ligament repair, PP block alone is inadequate for analgesia and requires a combination of IPACK or genicular nerve block. This combined blockade might achieve complete block of the deep sensory nerve and superficial sensory nerve of the knee without affecting lower limb mobilization, which is beneficial for postoperative rehabilitation [33, 34].

The present study has several limitations. First, the block duration may differ slightly in different sites of the peripatellar region. However, the pinprick sensation test of the entire peripatellar region for block duration measurement was not achievable. The use of a unified testing site at the anaesthetized skin in the patellar region to represent the entire peripatellar region could have greatly eliminated the bias. Second, the present study was performed in healthy volunteers. The pinprick pain may be different from the pain in real knee surgeries. Despite these differences, the PP block in our study covered the entire peripatellar region, and its expected analgesic characteristics may be similar to its actual analgesic characteristics after knee surgery. Further study will be conducted in this regard. Finally, the present study shares a basic limitation with most procedure-related studies, namely that it is not possible to blind the anaesthesiologist to group allocation. Although we assigned four anaesthesiologists to perform FN block and PP block separately, it may not have been sufficient to eliminate the potential bias of the unblinded anaesthesiologists. Although PP block might be beneficial for analgesia in knee surgery, it remains unsuitable for use in patients until sufficient clinical evidence has been obtained.

## Conclusion

It is feasible to identify and block the PP under ultrasound guidance. Compared with FN block, PP block provides noninferior cutaneous sensory block duration and completely blocks the area of the peripatellar region without affecting lower limb mobilization.

## Data Availability

The datasets used and/or analyzed during the current study are available from the corresponding author on reasonable request.
