# Correction: Motor-sparing peripatellar plexus block provides noninferior block duration and complete block area of the peripatellar region compared with femoral nerve block: a randomized, controlled, noninferiority study

**DOI:** 10.1186/s12871-022-01916-x

**Published:** 2022-12-09

**Authors:** Wen-Yi Gong, Chen-Guang Li, Jing-Yu Zhang, Xiao-Hui Liao, Cheng Zhu, Jie Min, Xiao-Fang Yue, Kun Fan

**Affiliations:** 1 Department of Anaesthesiology, Wusong Central Hospital, Shanghai, China; 2 Department of Anaesthesiology, First People’s Hospital of Tianshui, Gansu, China; 3 Department of Anaesthesiology, Second Hospital Affiliated to Lanzhou University, Gansu, China; 4 Department of Orthopaedics, Wusong Central Hospital, Shanghai, China; 5 Department of Neurology, Shanghai Sixth People’s Hospital, No. 600, Yishan Road, Shanghai, 200233 China; 6 Department of Anaesthesiology, Shanghai Sixth People’s Hospital, No. 600, Yishan Road, Shanghai, 200233 China

**Correction: BMC Anesthesiol 22, 334 (2022)**

**https://doi.org/10.1186/s12871-022-01863-7**

Following publication of the original article [1], the authors identified an error in Fig. 4. The correct figure is given below.

**Fig. 4:**
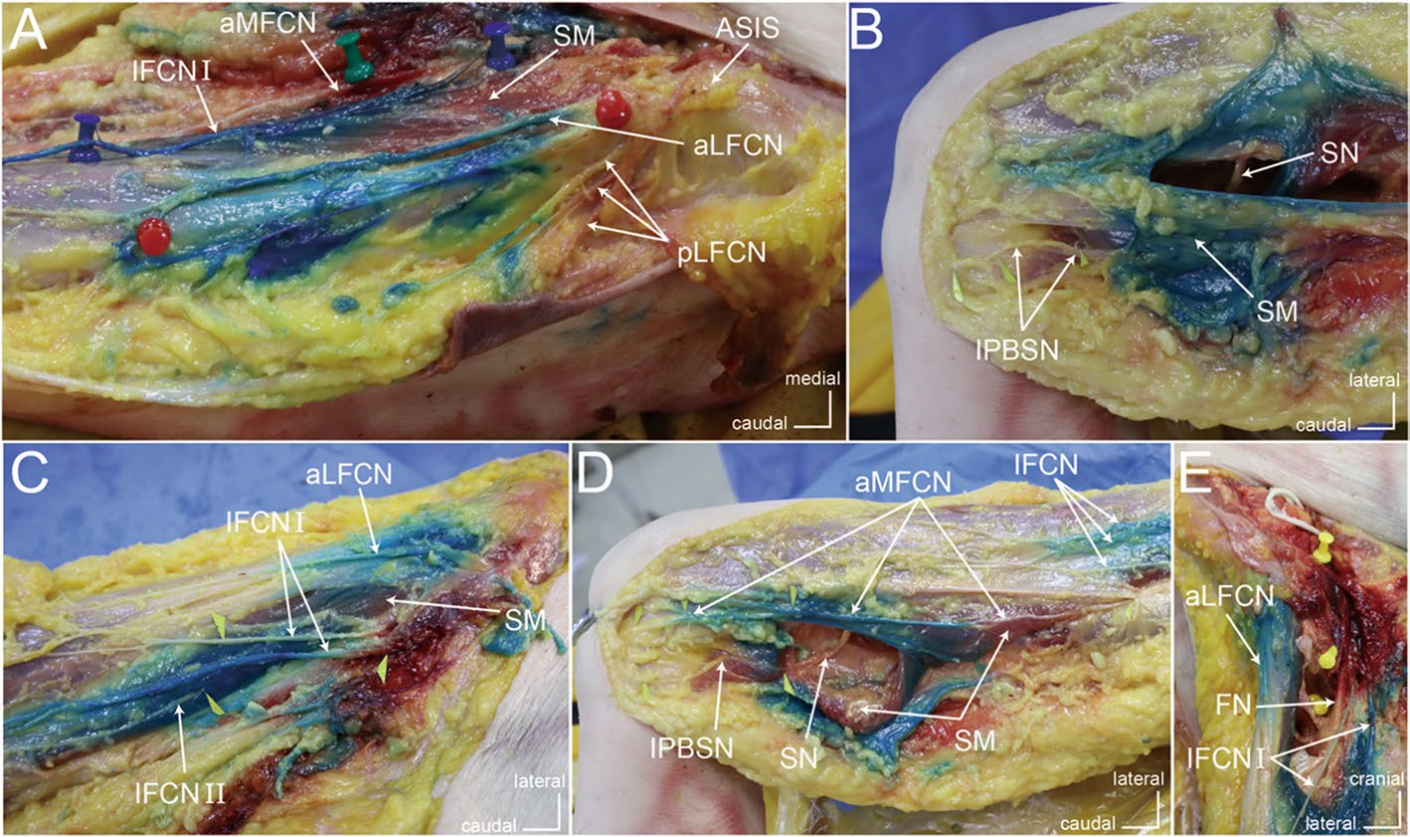
Course and staining of the peripatellar plexus 30 min after dye injection. **A** Course and staining of the LFCN; **B** Course and staining of the IPBSN; **C** Course and staining of the IFCN; **D** Course and staining of the aMFCN; **E** Staining of the FN. ASIS, anterior superior iliac spine; SM, sartorius muscle; aLFCN, anterior branch of the lateral femoral cutaneous nerve; pLFCN, posterior branch of the lateral femoral cutaneous nerve; IPBSN, infrapatellar branch of the saphenous nerve; IFCN I, intermediate femoral cutaneous nerve branch I; IFCN II, intermediate femoral cutaneous nerve branch II; aMFCN, anterior branch of the medial femoral cutaneous nerve; SN, saphenous nerve; FN, femoral nerve

The original article [1] has been updated.
